# A data management plan for the NESHIE observational study

**DOI:** 10.3389/fgene.2023.1273975

**Published:** 2023-12-06

**Authors:** Adéle Strydom, Jeanne Van Rensburg, Michael S. Pepper

**Affiliations:** Institute for Cellular and Molecular Medicine, Department of Immunology, and SAMRC Extramural Unit for Stem Cell Research and Therapy, Faculty of Health Sciences, University of Pretoria, Pretoria, South Africa

**Keywords:** sample, data, management, legislation, NESHIE

## Abstract

With regard to the use and transfer of research participants’ personal information, samples and other data nationally and internationally, it is necessary to construct a data management plan. One of the key objectives of a data management plan is to explain the governance of clinical, biochemical, laboratory, molecular and other sources of data according to the regulations and policies of all relevant stakeholders. It also seeks to describe the processes involved in protecting the personal information of research participants, especially those from vulnerable populations. In most data management plans, the framework therefore consists of describing the collection, organization, use, storage, contextualization, preservation, sharing and access of/to research data and/or samples. It may also include a description of data management resources, including those associated with analyzed samples, and identifies responsible parties for the establishment, implementation and overall management of the data management strategy. Importantly, the data management plan serves to highlight potential problems with the collection, sharing, and preservation of research data. However, there are different forms of data management plans and requirements may vary due to funder guidelines and the nature of the study under consideration. This paper leverages the detailed data management plans constructed for the ‘NESHIE study’ and is a first attempt at providing a comprehensive template applicable to research focused on vulnerable populations, particularly those within LMICs, that includes a multi-omics approach to achieve the study aims. More particularly, this template, available for download as a supplementary document, provides a modifiable outline for future projects that involve similar sensitivities, whether in clinical research or clinical trials. It includes a description of the management not only of the data generated through standard clinical practice, but also that which is generated through the analysis of a variety of samples being collected from research participants and analyzed using multi-omics approaches.

## 1 Introduction

Data management is important in any biomedical research project and facilitates the generation of high-quality and reliable data (Nourani et al., 2022). Broadly, a data management plan (DMP) may have the following benefits (Fadlelmola et al., 2021): 1) it protects the research participants and the project team; 2) it allows compliance with local data protection policies and legislation; 3) it maintains FAIR content; 4) it enables research that is transparent; and 5) it allows compliance with funder requirements. However, when considering a DMP, two key questions usually emerge. The first asks “what is a data management plan?”, while the second asks “how do you write one?”

In answering the first question, Stanford University defines a DMP as follows: “a written document that describes the data you expect to acquire or generate during the course of a research project, how you will manage, describe, analyze, and store those data, and what mechanisms you will use at the end of your project to share and preserve your data.”^1^ While some or all of these issues may have been considered when starting a research project, their documentation validates the DMP construction process. In so-doing, weaknesses in the plan are identified and a record is kept of what is proposed or completed. While potentially labor-intensive, the construction of a DMP is nevertheless viewed as a worthwhile exercise that addresses data management prior to the onset of a research project, rather than in a reactionary or improvised fashion during or towards the end of a project. The aim of any DMP should therefore be to focus attention on available resources and research infrastructure, and identify parties responsible for the inception, implementation and management of the DMP. The DMP should also highlight potential problems regarding long-term preservation and sharing of data and samples. When noting potential problems, some form of recourse or plan of action should accompany the DMP. This is because good data management can assist in preventing ‘bad’ research. While ‘bad’ research may result in the retraction of published papers, ‘good’ research provides data that is documented, stored, and includes reasonable routes for access.

Regarding the question on how to write a DMP, several online tools and questionnaires are available for this purpose (Fadlelmola et al., 2021). While questionnaires provide a guide to the nature of the data management issues that should be considered when writing a DMP, online tools include templates with information and guidance for ready-to-use DMPs. While these tools may be specific to a research project or funder, they typically include text that can be copied and pasted into a customized DMP. They also provide different export formats to support the requirements of funding applications. Examples of such online tools include DMPonline^2^ and DMPTool^3^. However, because research is discovery-oriented, the research process sometimes requires a change in direction and a revision of the intended data management path. As such, while the DMP should be constructed prior to the onset of the project, the DMP should also be viewed as a dynamic document that may be altered during the course of the research study. Every time the research plan changes, the DMP should be reviewed to make sure that it still meets the various regulatory and statutory requirements. This includes considering the funder-supplied set of policies and guidelines for data management and sharing (Fadlelmola et al., 2021). Funding bodies increasingly require that DMPs accompany study proposals when submitting funding applications. Importantly, such DMPs are typically required to consider open data sharing models (Wilkinson et al., 2016).

An increase in the need for open data sharing has resulted in the construction of a recommended set of standards for data management known as the “FAIR” principles. These principles have been defined as^4^.

• “*Findable*—Metadata and data should be easy to find for both humans and computers. Machine-readable metadata are essential for automatic discovery of datasets and services.”
• “*Accessible*—Once the user finds the required data, he/she needs to know how it can be accessed, possibly including authentication and authorization.”
• “*Interoperable*—The data usually needs to be integrated with other data. In addition, the data needs to interoperate with applications or workflows for analysis, storage, and processing.”
• “*Reusable*—The ultimate goal of FAIR is to optimize the reuse of data. To achieve this, metadata and data should be well-described so that they can be replicated and/or combined in different settings.”

While data management planning may have technical challenges that include not clearly knowing the benefits and best practices for a research project at inception (Lefebvre et al., 2020), the practical implementation of FAIR principles in low- and middle-income countries (LMICs) may be hindered by a number of additional factors (Fadlelmola et al., 2021). These may include a lack of research funding, inadequate human resources, limited research data management guidelines and policies, a lack of training in research data management, inadequately secure and/or reliable technology, inefficient or inadequate archiving of data, and inefficient support from academic institutions regarding data management. Because of historical, cultural, and ethical concerns, special consideration should also be made in the construction of DMPs when planning to share African-centric data.

Nevertheless, the data management process not only consists of creating study-associated documents such as data sheets or case report forms (CRFs) and consent forms, but also involves training of the research team, creating databases, capturing and validating data, managing data discrepancies, resolving data disagreements, describing the processes of data coding and extraction, access control, recording the data management process, and providing security throughout the duration of a research project (Nourani et al., 2022). When working with databases, electronic data management systems require sufficient hardware, software, communication technologies, policies/guidelines for data collection, quality control of data, and security in order to be operational (Nourani et al., 2022).

Regardless of the form the DMP assumes, the basic principles that govern its construction include the preservation of and (continued) access to the research data (Dunie, 2017). This not only ensures the reproducibility, traceability, and reliability of the research data, but also assists in reducing the costs of performing additional research investigations (Williams et al., 2017). Reproducibility and traceability are at risk when robust policies and documentation regarding data management are absent. As such, it is important that issues regarding data sharing and secondary use of data are covered in the DMP, especially in relation to cross-border sharing of samples/data. In most instances, a material transfer agreement (MTA) or data transfer agreement (DTA) between collaborators will resolve data and/or sample transfer issues. Such agreements may be listed in the DMP. Globally, funders and research institutions are promoting open science policies and practices to manage research data (Lefebvre et al., 2020).

Since nearly 50% of medico-legalcases brought against the South African National Department of Health between 2019 and 2020, which totaled ZARR53-billion, were linked to birth asphyxia, neonatal encephalopathy and cerebral palsy, it is important that the management of data linked to any or all of these conditions are carefully considered and well documented.^5^ ^,^ ^6^ ^,^ ^7^ This paper will consequently describe the general principles used to design and structure the data management plan for the national multi-institutional NESHIE (Neonatal Encephalopathy with Suspected Hypoxic Ischemic Encephalophathy) project being overseen by the Institute for Cellular and Molecular Medicine (ICMM) in the Faculty of Health Sciences at the University of Pretoria (UP), South Africa, in collaboration with the Universities of Cape Town (Cape Town, South Africa), Stellenbosch (Cape Town, South Africa), and the Witwatersrand (Johannesburg, South Africa).

Within this ongoing study, a ‘multi-omics’ approach is being employed to identifying proximal biomarkers and increase understanding of the pathogenesis of NESHIE in a highly vulnerable population. This study is unique in that genomic, epigenomic, transcriptomic, proteomic and metabolomic (‘multi-omic’) analyses are being performed on the same individuals on whom large-scale clinical data (up to 1,500 variables per neonate-maternal pair) is being collected. This data includes imaging information (cranial ultrasound and limited magnetic resonance imaging data), placental pathology data, and an additional molecular component investigating the potential pathomicrobiome associated with placental tissue samples. While a full contingent of samples is not necessarily collected for every participant, a DMP that comprehensively describes the management of the clinical and multiple molecular data outputs was, and remains, necessary for the NESHIE study. However, at the onset of sample and data collection for the NESHIE study in 2019, existing DMP templates did not fully capture the data management needs of the study. A detailed DMP was subsequently developed and is presented here for use in research projects or clinical trials involving NESHIE or associated conditions, particularly in LMICs, for clinical research investigations involving multi-omic data outputs, and/or investigations involving vulnerable populations. Importantly, this DMP was constructed in the context of South Africa and was therefore guided by the requirements of the National Health Research Ethics Council (NHREC), the Protection of Personal Information Act 4 of 2013 (POPIA), and the National Health Act 61 of 2003 (NHA) and the Declaration of Helsinki. It was additionally guided by Good Clinical Practice (GCP) principals. It describes the safe, secure and ethical manner in which clinical and multi-omic sample-associated data collected from vulnerable populations may be collected and shared in a research team or amongst collaborators.

The NESHIE study DMP template, which is available as Supplementary Material S1, is user friendly, easy to access and can be adapted to most research projects or clinical trial. To our knowledge, this is the first data management plan of this nature to be published.

## 2 Framework of the NESHIE data management plan

The NESHIE Data Management Plan consists of a combination of DMP templates with sections relevant to a wide range of clinical data and associated molecular analyses outputs generated as part of the study.^8^ ^,^ ^9^ ^,^ ^10^ Sections have been modified to the requirements of the study, thus creating a living document that is easy to maintain. The NESHIE study DMP complies with the policies and guidelines of all stakeholders (academic institution and funders) involved in the project and has been approved by the University of Pretoria Research Ethics Committee (REC; reference number 481/2017).

**FIGURE 1:**
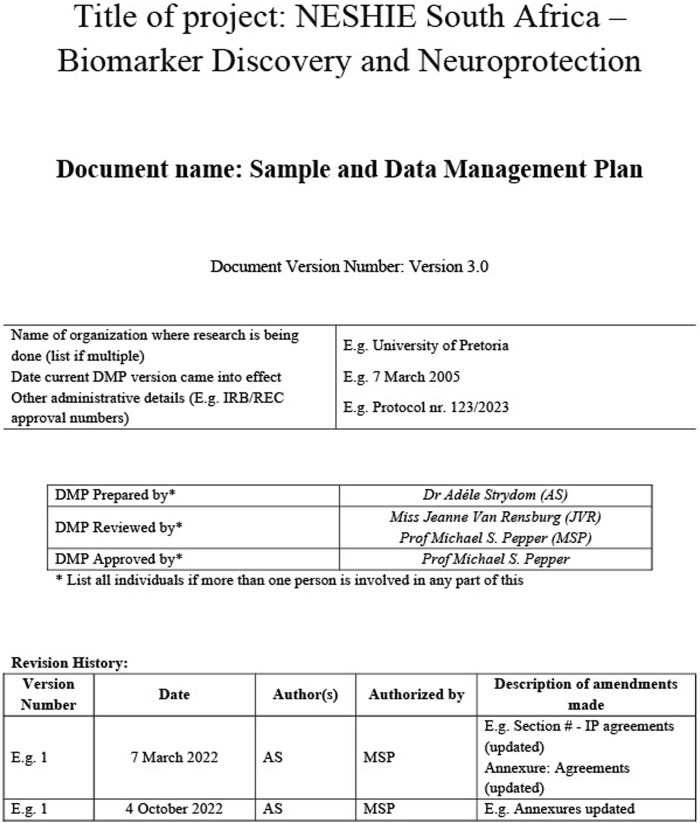
The cover page for the NESHIE Data Management Plan. Image created by AS.

The first page of the DMP template consists of a cover page (Figure 1) followed by the Table of Contents on the next page. In the case of the NESHIE project, a project manager and a DMP coordinator review the document regularly. This process considers amendments made to the study protocol, SOPs, and local and international policies/regulations. These amendments are listed on the cover page of the DMP under ‘Revision History’ (Figure 1) and indicates the dates and details of the changes and by whom they are made.

The sections that follow the Title page in the NESHIE DMP include.

• A *List of abbreviations* that is specific to the NESHIE project;
• *Definitions* of terms used in the DMP that may otherwise be misinterpreted or misunderstood;
• The *Scope* of the DMP that briefly describes the policies and regulations to which the NESHIE project complies, and explains the application of the DMP; and
• A short *Protocol summary* or *Introduction* that provides a broad overview of the NESHIE study protocol and that may include the variables used to analyze critical data.

**FIGURE 2:**
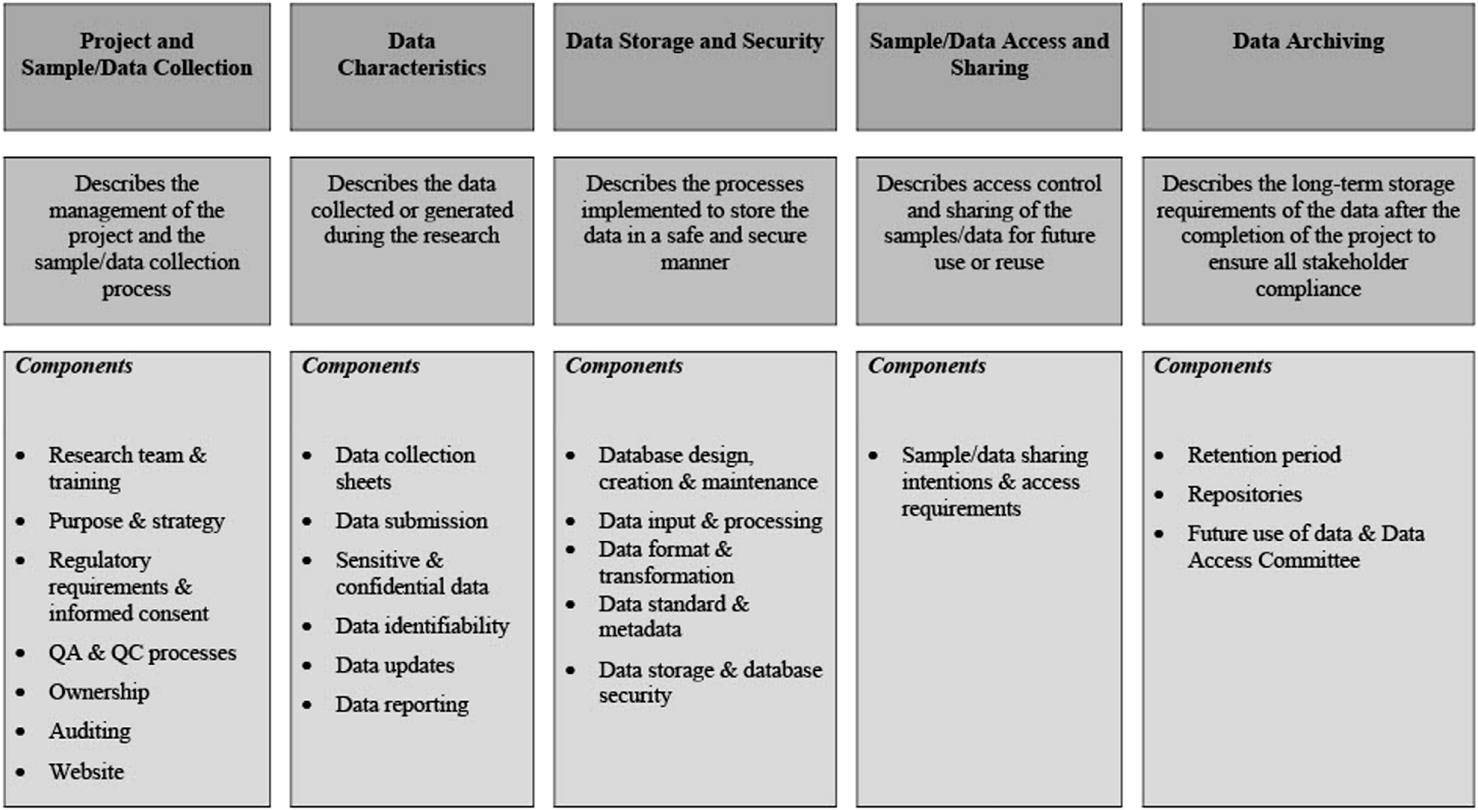
Core elements as described in the NESHIE data management plan. Image created by AS.

The points above form the foundation of a DMP and should provide the reader with sufficient background information to facilitate an understanding of the content of the remainder of the DMP.^8,9,10^ As summarized in Figure 2, the core elements of a DMP are then described in more detail in the subsequent sections. While the NESHIE study information has been used for this purpose, the details should be amended to suit the needs of each study being performed.

### 2.1 Project and sample/data collection

#### 2.1.1 Research team and training

A comprehensive DMP includes all role players and organizations, and describes their roles and responsibilities (Michener, 2015). These responsibilities may include collection and entry of data, quality control, creation and management of metadata, submission of data to an archive, and the administration of databases. Furthermore, the research team and training section of a DMP (1) specifies the research team responsible for collecting data and samples at the participating study sites, and (2) describes the project-specific training requirements for the research team.

Table 1 summarizes the roles and responsibilities of the NESHIE research team, and Table 2 describes the NESHIE training requirements for the study team members. Importantly, while this may not be a formal requirement for all studies, given the long-term aspirations of the research associated with the NESHIE study to transition into a clinical trial, Good Clinical Practice (GCP) training is required by all medical officers/staff and research assistants/associates involved in sample and data collection. Renewal of GCP certification is also required. Each study will however have its own requirements regarding GCP training, including identification of those team members who require certification. In relation to the NESHIE study, further training is provided prior to study onset for the following:

• Study protocol;
• Study annexure documents;
• Consent documents and associated process;
• Sample collection; and
• Data collection and capturing to electronic platforms.

**TABLE 1:** Examples of roles and responsibilities of the NESHIE research team.

	Project roles	Project responsibilities
Project leads	Principal Investigator	• Project management team at academic institutions and study sites
	Project Manager	
	Lead Neonatologist	
	Lead Placental Pathologist	
	Lead Obstetrician	
Project site support	Site Neonatologists	• Management of sample and data collection from research participants at participating study sites
	Site Placental Pathologists	• Management/Co-ordination of neurodevelopmental follow-up with research participants at study sites
	Site Obstetrician	
Biomarkers and clinical trial components	Principal Investigator	• Imaging biomarkers of research participants (E.g. MRI scans)
	Project Manager	• Analyses of biological samples (E.g. genomic/transcriptomic/metabolomic/proteomic and placental microbiomic analysis)
	Radiology/Imaging team	• Clinical trial protocol development
	Molecular analysis team	
	Clinical trials team	
Electronic data capture platform	Bioinformaticians	• Administration and maintenance of electronic data capture platforms (E.g. REDCap)
	IT support	
	Data Manage	
Study appointees	Medical officers and clinical support staff	• Collection, storage and transport of samples
	Scientists	• Data collection and capture
	Bioethicists	• Quality control of data
	Statisticians	• Molecular data analysis
	Administrative staff	• Radiography and imaging
		• Sample and data management
		• Clinical trial management

**TABLE 2:** Examples of study training requirements.

Training document	Who receives the training	When is training received
Protocol documents	All study members	Prior to study onset
Informed Consent Documents & Associated Process	All study members	Prior to study onset
SOP documents	All relevant study members. Not all study members may require training on all study-associated SOPs. If necessary, SOP training should be segmented according to the respective study roles and responsibilities	Prior to study onset
Good Clinical Practice (GCP)	All clinical and scientific study members. All relevant administrative study members	Prior to study onset; renewable every 2–3 years
Good Laboratory Practice (GLP)	All laboratory-based study members	Prior to study onset; renewable every 2–3 years
Case Report Form Completion & Data capturing	All data-associated study members and project site support team	Prior to study onset

Training typically starts at the initiation of the study at each site but may be ongoing. Additional training may be arranged as required or requested by study associates. Training is recorded during the formal training sessions. As best practice, these records should be retained in a study-associated logbook or file for the full duration of the project, as is the case for the NESHIE study.

#### 2.1.2 Purpose and strategy of sample/data collection

**FIGURE 3:**
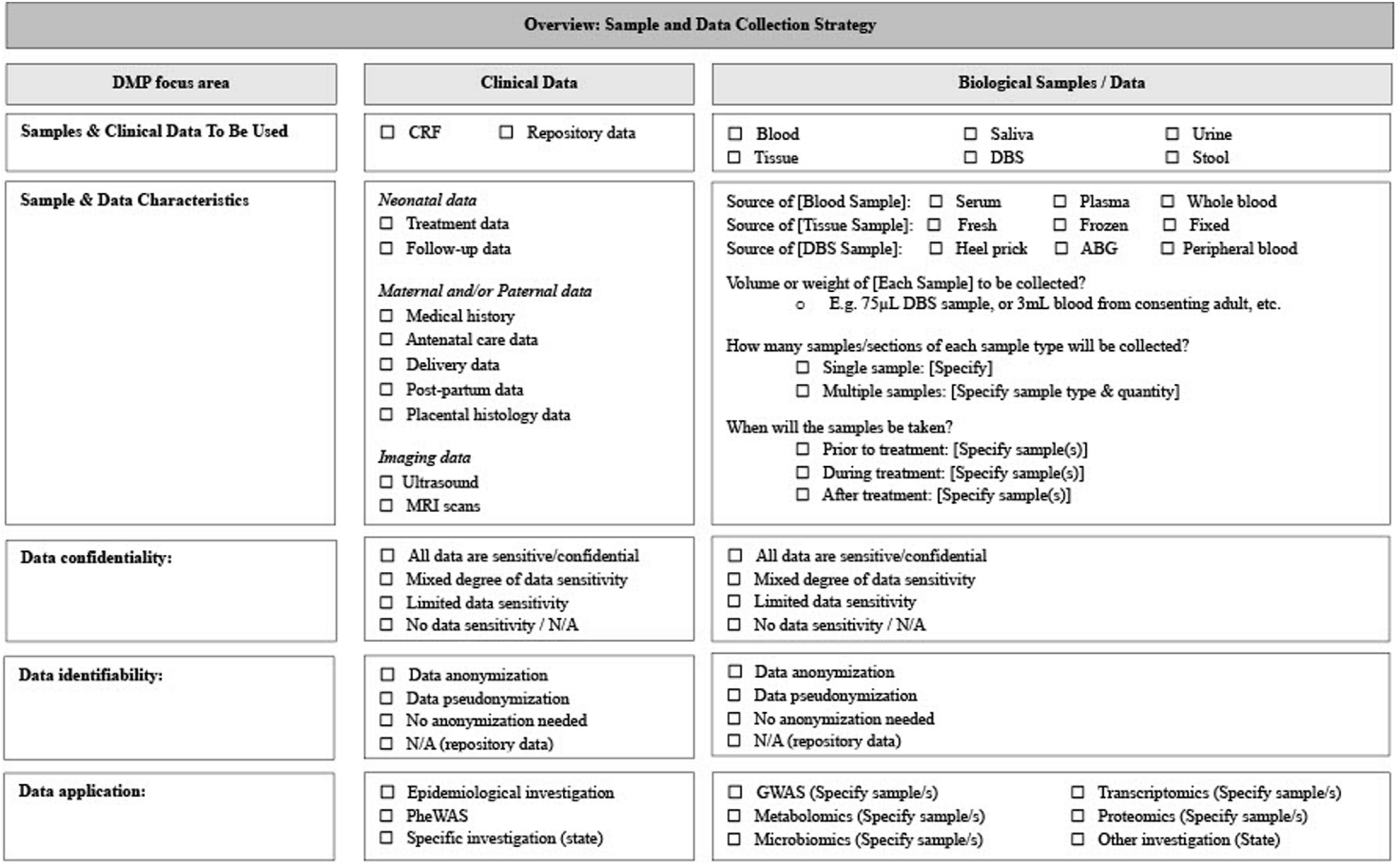
Sample and data collection summary template. Image created by JVR.

A DMP will include information that explains what samples and data are to be collected (Michener, 2015). Typically, a list of various types of samples/data that are expected to be collected or created, which could include biological samples, patient records, or images, will be provided within a DMP. The source of the samples/data is usually also provided. In this section, the NESHIE study DMP outlines what samples and data should be collected/generated and what the collection and future analysis strategy regarding clinical and molecular data is. This information is summarized in Figure 3. Importantly, while Figure 3 provides an overview of the samples and data collection purpose, collection strategies are described in detail following the overview. For the NESHIE study, this includes providing details on the collection of time-sensitive samples and data (e.g., umbilical cord blood, dry blood spot samples, and follow-up data at 9–12 months of age), as well as samples and data unaffected by collection time (e.g., peripheral blood and baseline laboratory values). This is important to provide for context given the need to account for multiple sets of data generated for the same patient at different points in time. For example, umbilical blood will result in the generation of both genomic and transcriptomic data, while dry blood spot samples allow both metabolomic and proteomic datasets to be generated at two different timepoints within 3 days of life.

Lastly, within this section of the DMP, it is also important to state the aims and objectives of the sample and data collection process for context. The aims and objectives were summarized in the NESHIE DMP as follows:

1) Providing a detailed description of grade 2–3 NESHIE in South African tertiary-level hospitals;
2) Biomarker identification; and
3) Determining whether genetic factors are associated with and potentially contribute toward the presentation of NESHIE.

The following additional long-term aims are included with biomarker identification:

1) Predicting susceptibility to NESHIE;
2) Identifying factors other than hypoxia/ischemia that may cause/contribute to the clinical presentation;
3) Quantifying the extent and duration of hypoxia if present; and
4) Defining prognostic factors used to predict the medium-to-long term consequences in affected neonates; thus minimizing risk by increasing awareness and altering management during pregnancy and the peripartum period.

#### 2.1.3 Regulatory requirements and informed consent

Many funders require that researchers receive prior approval from their institutional ethics committees before the submission of a grant proposal and before the start of the actual research (Michener, 2015). The NESHIE study is no exception. Ethics approval was obtained from the UP REC (primary ethics committee) and all other participating institutions for the NESHIE project. Permission was also obtained for the research to be conducted at the respective institutions included in the NESHIE study. This information needs to be recorded in the DMP. While the NESHIE study described this information using bullet points, this information can also be tabulated. The regulatory body’s name, study approval number and approval date should be noted. If additional approvals are required for the purposes of a study, for example, hospital or internal review committee approvals, the DMP should capture this information as well.

However, since study documents may require amendment during the course of a study, it is also important that a record of changes to study records be kept. While this data may not necessarily be reflected within a DMP, reference to its storage location should be made, as is the case for the NESHIE study DMP. An example of a document tracking log is reflected in Table 3.

**TABLE 3:** Example of a document tracking log.

Document amendment no.	Document name	Document version in use under current amendment	Date of IRB/REC amendment submission	Date of IRB/REC amendment approval
Amendment 1	Study Protocol	Version 1 (Original version)	No changes to submit	N/A
Amendment 1	Study SOP: Sample collection	Version 2	27 March 2023	07 May 2023

Depending on funder or regulatory body requirements, it may be necessary to explicitly state what the inclusion and exclusion criteria for participation in a study are. While this is the case for the NESHIE study, the DMP may also simplify this by providing a reference to the relevant study document. These details may be provided in text or table format and are at the discretion of the author of the DMP. This information is often provided in order to contextualize the informed consent process since ethics approval requires that informed consent be granted by research participants, that data are de-identified, and that data access and use is restricted.

**FIGURE 4:**
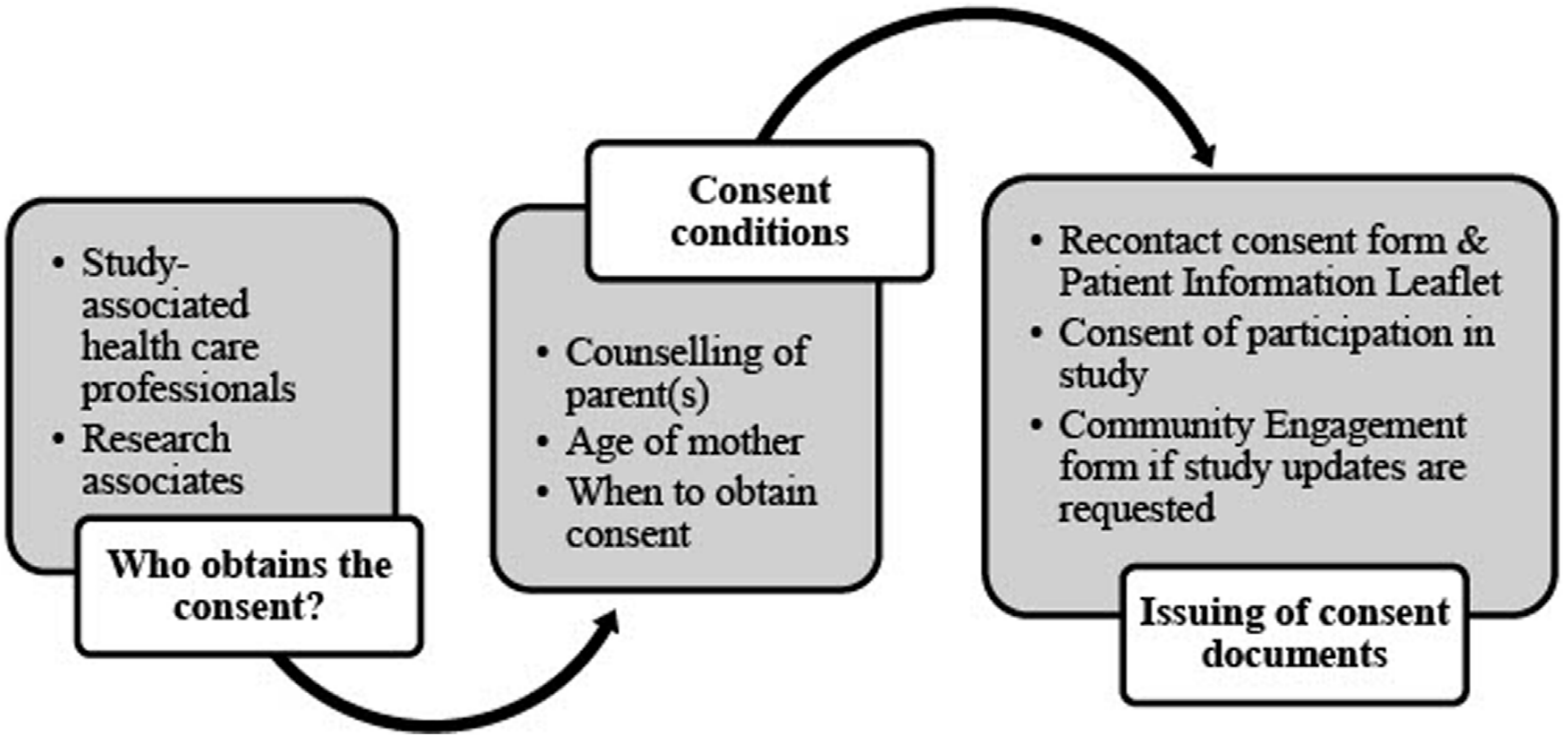
Informed consent process in the NESHIE study. Image created by JVR and AS.

An informed consent form outlines the terms of research participation and may include or exclude future usage of data (Hardy et al., 2016). Researchers may not re-analyze research data in any form when the informed consent form excludes the future and unrelated usage of that data. In such cases, new applications must be submitted instead of amending current ethics approvals. The DMP needs to refer to the consent documents and where they may be found. Similarly, it describes the consent process and associated vetting thereof. This includes the quality control process in ensuring the validity of each participant’s signed consent form(s). The informed consent process is explained in the NESHIE DMP and summarized in Figure 4.

#### 2.1.4 Quality assurance and quality control processes

Quality assurance (QA) and quality control (QC) processes measure, assess, and improve the quality of data, software and other study-related factors. According to the nature of the study and degree of research funding, specific QA/QC guidelines may have to be followed (Michener, 2015). It is however good practice to provide a description of the QA/QC measures employed in a research project, which may involve training, the calibration and verification of instruments, and double-blind data entry. It is critical to state who will be responsible for performing the various QA/QC tasks, when they will be performed, how frequently they will be performed, what potential problems are expected and what contingency plans are in place for this. If templates are used for the purpose of QA/QC and not already included in the protocol, these should be reflected in the DMP and adjusted according to the needs of the study (as is the case for the NESHIE study).

**FIGURE 5:**
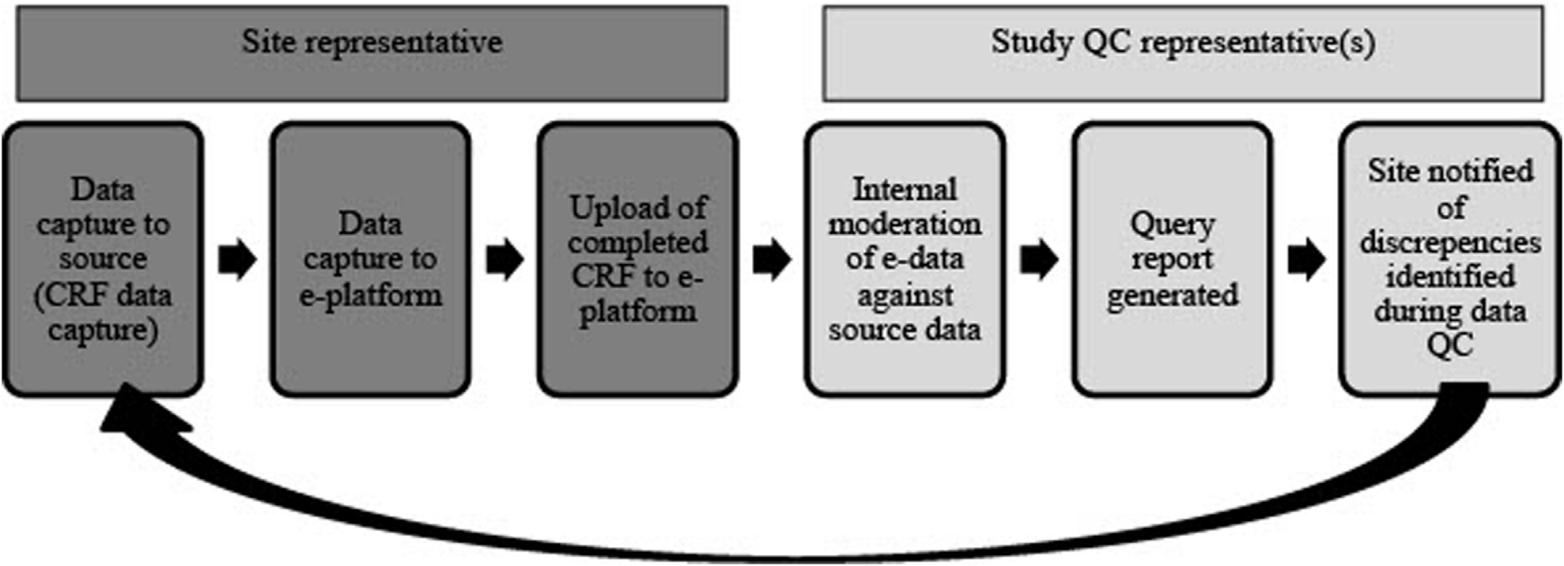
An overview of the QC process for study-associated NESHIE data. This review cycle is repeated until all data queries are cleared and data are validated as accurate. Image created by JVR.

Quality control (QC) reports for the NESHIE study are done manually or by using REDCap’s Data Resolution Workflow which is an inbuilt data query function in REDCap (Figure 5). QC for placental histology and de-identified scans/images are conducted using standardized report forms from blinded individuals. This is done to account for any inter-observer bias. Unblinded QC is performed for informed consent documents and other core anonymized clinical data (e.g., severity grading) using standardized report forms.

#### 2.1.5 Ownership

The DMP must indicate ownership of the data (Fadlelmola et al., 2021). According to Thaldar et al. (2022), ownership in law implies a relationship between the owner and the object (or thing) in respect of which the owner acquires certain legal rights and entitlements. Neither a hospital site nor a research project can acquire, exercise, or enforce any of the rights contemplated in terms of legal ownership. As a consequence, NESHIE research data would belong to the Principal Investigator (PI), Prof Michael S. Pepper, as prescribed by Thaldar et al. (Thaldar et al., 2022).

Intellectual property including Material Transfer Agreements between collaborators and the NESHIE study are also mentioned in the DMP and are listed in the Annexure but retained separately to the DMP owing to confidentiality. Commercialization has been considered, but as the study has not yet reached a stage where this is relevant, a specific framework for commercialization has not yet been developed. Funding agencies for the NESHIE project are clearly indicated. Publication outputs are reflected in the NESHIE study DMP on a regular basis.

Because the NESHIE study is a collaborative project, all collaborators and their roles in the study are provided in the DMP. This list can be updated as additional collaborators join the study and includes the following:

• all hospital sites where participants will be recruited;
• participating academic institutions and their associated clinical facilities and representatives;
• collaborators assisting in establishing systems for sample processing and sample quality control protocols;
• national and international centers where genomic, transcriptomic, proteomic and metabolomic data will be processed and analyzed; and
• collaborators assisting with MR imaging.

#### 2.1.6 Auditing

The DMP should include the details of an audit plan for a clinical trial, or in the case of a research project, refer to the relevant SOPs. Since it is an observational study, the NESHIE study does not have a formal audit plan as stringent as what would be found in a clinical trial. More specifically, auditing for the NESHIE study is performed internally, rather than by auditors/monitors contracted externally to the study. Nevertheless, auditing of the data is a critical component of the study to ensure that data of the highest quality is reported in the public domain. As such, auditing of the data ties closely to data QC processes and can be viewed as a two-step process. Firstly, a data manager will ensure that data captured to case report forms is concordant to what is captured to the study’s electronic data capture platform. Where discordance or data missingness is noted, data queries are raised for the study site to attend to until data concordance is observed. Once data is concordant across sources for each section of data, the data section (‘instrument’) is locked. This first step of data validation/auditing is performed on a continual basis. The second phase of data auditing involves senior members of the research team (project manage and molecular data manager) who review data from all locked instruments. This involves several point and cross-sectional data checks. If no further data errors are noted, the participant record is locked in entirety. The second phase of data auditing is largely dependent on the complete review and locking of data instruments and therefore occurs as needed at variable intervals. In addition, audit trails are automatically generated through the various electronic data collection platforms used as part of the NESHIE study, are downloaded on a weekly basis by the data manager, and retained on an independent local server where periodic checks for completeness are conducted by the project manager. Spot checks of study records are also conducted by the project manager to ensure that enrolled participant’s data records are complete. Each study will need to tailor its data auditing needs according to its aim(s), objectives and long-term aspirations.

#### 2.1.7 Website

The DMP should provide details of a website if one has been created for a research project or clinical trial. A website for the NESHIE project is under development and is aimed at providing general information on HIE, for example, as well as current research and publications.

### 2.2 Data characteristics

#### 2.2.1 Data collection sheets

A brief description of the data/sample collection sheets (case report forms or CRFs) should be provided in a DMP. This may include the development of the collection sheets, general guidelines for the completion of these collection sheets, amendments and recordkeeping practices. The NESHIE data collection sheets have been designed by experts. For example, neonatologists have developed and vetted the neonatal data collection sheets, while obstetricians have developed and vetted the maternal and obstetrics data collection sheets. The design was based on local and international best practices and within the South African context where applicable. Guidelines for the completion of the data collection sheets are provided in the NESHIE study documents, during training of the research team, and/or within the REDCap database. A list of all amendments to the design of the data collection sheets should be submitted to an IRB/REC prior to enforcement, with redline copies kept for the study’s recordkeeping purposes. This record is maintained by the project manager of the NESHIE study. An electronic record is also maintained of all the CRF versions.

#### 2.2.2 Data submission

A DMP for a research project or clinical trial should describe how the data is submitted, whether it is done manually or electronically, or even both. This should also include the tools being used to generate the data, for example, standardized case report forms and electronic data capture platforms (eDCPs).^8,9,10^ The mode through which data will be submitted should be compliant with the various guidelines and policies applicable to the research being conducted. For example, if performing a clinical trial and enforcing GCP guidelines, it is necessary to have access to the source documents.^11^ In clinical settings, this often relates to accessing hospital records. However, if obtaining hospital records (source documentation) is challenging, the DMP should provide a clear set of instructions on when and how photocopies of the hospital records should be made for the research site’s reference and for auditing purposes.

In the NESHIE study:

• all clinical data are captured manually on data capture sheets or CRFs and these serve as the source documents;
• de-identified electronic copies of the source documents are then uploaded onto REDCap; and
• documents with identifying information necessary for QC purposes are uploaded to the study-associated server using LogicalDOC.

Data verification is done at the hospital site where information is captured onto the CRFs and when data are captured onto the eDCP(s). Each NESHIE participant’s record is linked to an electronic copy of the CRF. This includes capturing the barcode links affiliated with the specific samples that were collected from each consenting participant and sent for long-term storage in an accredited biorepository. Long-term storage of NESHIE samples is undertaken by Clinical Laboratory Services (Braamfontein, Johannesburg, South Africa) which is a highly reputable biobank in South Africa. This process, which includes the means through which Clinical Laboratory Services captures barcoded sample data to its laboratory information management system and disseminates it, is detailed across the NESHIE DMP and protocol. A simple template that can be modified and used for the purposes of capturing this information is reflected in Table 4.

**TABLE 4:** Tools used to generate and submit data in the NESHIE study.

Generated Document/Data	Submission
Informed Consent Forms (ICFs)	☐ Electronic: Site and Study records
	☐ Hard-copy: Site records
Community Engagement Form(s)	☐ Electronic: Site and Study records
	☐ Hard-copy: Site record
	☐ Hard-copy: Study Master File
Clinical data storage	☐ Electronic: Site and Study records
	☐ Hard-copy: Site records
	☐ Hard-copy: Study Master File
Basic data for sample storage	☐ Electronic: Sample storage facility (E.g. biorepository)
	☐ Electronic: Site and Study records
	☐ Hard-copy: Site records
	☐ Hard-copy: Study Master File
Molecular data storage	☐ Electronic: Sample analysis facility
	☐ Electronic: Site and Study records
	☐ Hard-copy: Site records
	☐ Hard-copy: Study Master File

#### 2.2.3 Sensitive and confidential data

Data sensitivity should be covered in the DMP and an explanation should be provided about how it will be treated (Hardy et al., 2016; Fadlelmola et al., 2021), for example, compliance with research institutional policies or national legislation. It is therefore mandatory that researchers who collect and analyze data should have training on ethical practices which include confidentiality, informed consent and data protection (Hardy et al., 2016). Sensitive data from vulnerable communities require more stringent guidelines. Thus, when a research collaboration becomes large and complicated, the project leads or principal investigators may require multiple approvals for just one project (Hardy et al., 2016). A DMP should include clear instructions on how sensitive or confidential information will be collected, protected and used.

Within the NESHIE study DMP, all clinical and molecular data are considered to be confidential due to the vulnerability of the research participants (mothers and infants). Where access to sensitive information is required for the purposes of QA/QC, the DMP clearly indicates the responsible parties for these processes. These processes describe how, when, and the frequency with which this information will be accessed. Similarly, to prevent the identification of individuals, aggregated research findings are reported.

#### 2.2.4 Data identifiability

Participant anonymization is a requirement for all research studies and clinical trials involving human participants when releasing data into the public domain. The protection of research participants’ data necessitates constant supervision. Therefore, when a coding system is used to ensure participant anonymity for this purpose, this must be explained in the DMP. Several methods for participant anonymization are available; Rodriguez et al. (2022) provide an excellent review of the many methods used for this purpose.

All research participants in the NESHIE study are assigned a random alphanumeric code at the time of participant screening to protect their privacy. This unique identifier remains unchanged throughout the entirety of the study and is applied equally to clinical and sample-associated data. Individual clinical data are not publicly released, however aggregated data (a form of *k-*anonymization) is consensually made publicly available through journal publications to further protect participants identities. Additional sample anonymization is applied once samples are deposited in a biobank. Regarding sample-associated data, while identification of research participants is very low with proteomic, metabolomic, and transcriptomic data, it is not possible to guarantee the absence of re-identification with genomic data. Nevertheless, metadata for sample analysis in the NESHIE study is typically limited to variables such as sample source (e.g., heel prick), time of collection relative to the time of birth, disease severity and the receptacle(s) used for sample collection (e.g., Whatman 903 protein-saver cards).

However, it is important to note that during the data/sample collection process, some members of the research team will have full access to personal/identifying information. Such detail must be clearly explained within the DMP. In the NESHIE study, for example, this will include those individuals responsible for collecting data from hospital records, as well as those responsible for ensuring that placental histology slides are obtained as part of the study’s QC procedures. As such, each site is expected to maintain a site-specific master list which links patient information to the Study IDs. These lists are not stored at the central database level for the purposes of the NESHIE study but must be retained at site-level and maintained at all times. Nevertheless, most members of the team will have access to pseudonymized information in order to fulfil their various roles and responsibilities.

#### 2.2.5 Data updates

The DMP should explain how data will be updated or become redundant when revisions are made and subsequent CRF versions are produced. This also applies to the NESHIE study; the DMP is revised and updated when necessary. Although some data may become redundant when revisions are made, information is retained from any system that is updated for recordkeeping and backup purposes. All changes made on the NESHIE study documents or REDCap database are reflected in the amendments submitted to the REC. A record is kept of all changes made on the NESHIE study documents while data dictionaries are kept that reflect all changes made on the different REDCap versions. This process is explained in the DMP.

#### 2.2.6 Data reporting

The requirements and frequency of data reporting should be described in the DMP, and where feasible, should be reflected in the study-associated timeline. In the NESHIE study, the data reporting requirements start when a potential participant is screened for inclusion. Data is reported after validation through the entire QC process and only fully validated data is published. Internal reporting regarding screening, enrolment and sample collection is typically done monthly to the PI but may be adjusted according to study needs. Data obtained from sample analyses is reported on an ongoing basis when sufficiently validated.

### 2.3 Samples/data storage and security

#### 2.3.1 Database design, creation and maintenance

**FIGURE 6:**
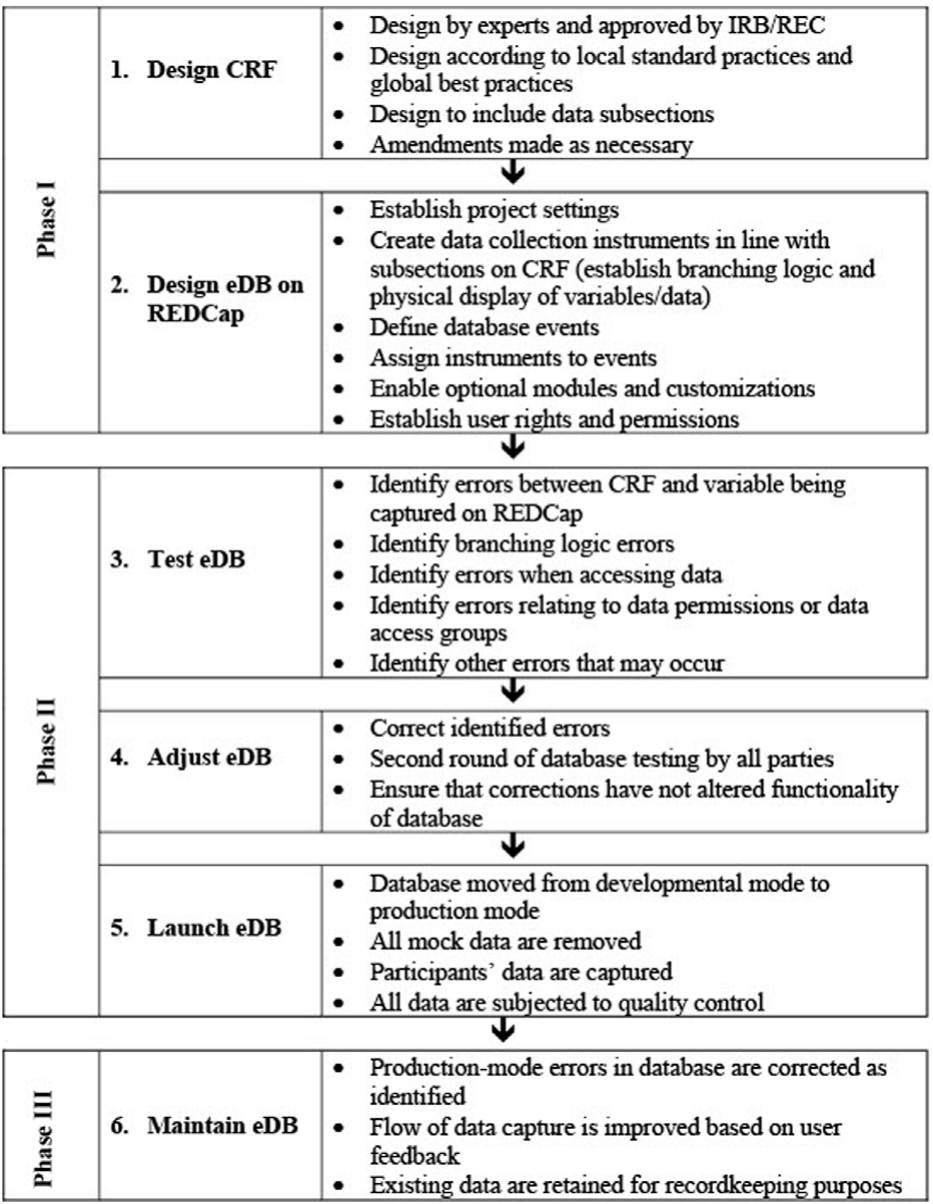
The overall flow of database creation and maintenance in the NESHIE study. Image created by JVR and AS.

The process involved in establishing an electronic database platform should be described in a DMP, starting with the requirement analysis, conceptual, logical and physical design, until the launch and maintenance of the database(s). Figure 6 summarizes the process for the establishment of the REDCap database for the NESHIE project.

#### 2.3.2 Data input and processing

Guidelines for data entry and data processing should be explained in the DMP. In the NESHIE DMP, data entry and processing are described with regard to the CRFs and the REDCap database. Data entry on the CRFs is guided through instructions on the forms and through regular training of research associates. The REDCap database provides prompts that guide the data input. Data anomalies or missing data are managed through the QC process. A single data entry method is used with on-site and off-site verification of data. Hospital site clinical appointees make corrections on the paper documents and the data manager makes changes on the electronic data. The data manager also keeps records of the data logs/audit trails on REDCap and changes made to the CRFs. Data generated through the NESHIE study is not linked to external/third-party databases except where expressed consent is provided for this (e.g., submission of molecular findings to a data repository), or where it is necessary as part of the sample/data analysis process (e.g., analysis of MR images).

#### 2.3.3 Data format and transformation

As technology changes, some current data and file formats may become obsolete (Michener, 2015). Therefore, a good choice of data and file formats includes those that are non-proprietary and commonly used within the associated research field, for example, comma separated values (CSV) as a replacement for Microsoft Excel^®^ formats (.xlsx). Data formats should be consistently applied throughout the duration of the study and must also be considered in relation to archiving conditions. The study-related data formats must be disclosed in the DMP. An example of the data formats used in the NESHIE study, as described in the DMP, is found in Table 5.

**TABLE 5:** Examples of clinical, metabolomic, genomic and imaging data inputs and formats for the NESHIE study.

DATA	Software used	FILE formats
Clinical	• SPSS Statistical Software	.csv and.xlsx files
	• Stata Statistical Software	
	• SAS Statistical Software	
	• R Statistical Software	
Metabolomic	• Liquid Chromatography-Mass Spectrometry data files	.csv and.xlsx files
Genomic	• Illumina short read sequencing	FASTQ
	• BGI-based sequencing platforms	
Imaging	• MRI: Hyperfine Cloud Picture Archiving and Communication System (PACS) or local hospital PACS	• MRI: DICOM files
	• CUS imaging software	• CUS: .jpeg files

In the NESHIE study, while some molecular data may undergo file format transformations, most of the file formats are accessible in common analysis platforms and are accessible for prolonged periods of time. As such it is important to note that data export options must be reflected within the DMP. Data export should be considered relative to the export for internal review of data, as well as external data review/sharing (as is the case for repositories). Personal health information should be removed from all datasets where authorization to view such data has not been granted and would constitute unethical behavior if shared. Data export will be covered in more detail in a subsequent section.

#### 2.3.4 Data standard and metadata

According to Michener (2015), “metadata are the details about what, where, when, why, and how the data were collected, processed, and interpreted”. Metadata allows for the discovery, use, and the accurate citation of data and files. It explains the names, structure, and storage of data and files and details the research environment, experiments, and analyses. A good documentation strategy includes the following three steps (Michener, 2015; Fadlelmola et al., 2021): First, identify the types of information that must be collected which will allow the discovering, accessing, interpretation, usage, and the citation of data. Second, determine whether a community-based metadata standard should be implemented, for example, ICD-11 (International Classification of Diseases, 11th ed). This may be required by a data repository, archive, or domain professional organization. Third, identify software that can generate and manage metadata content. As such, a DMP should adequately describe how the data will be formatted and standardized for present and future use, whether or not a data dictionary is available (if REDCap or similar electronic data capture platform is being used), whether file naming conventions are being applied (e.g., HGNC ID for naming of genes)^12^, whether existing metadata is sufficient for data interpretation, how CRF versions and associated data will be tracked across data records, and whether data will adhere to FAIR principles.

In the NESHIE study, formatting of variables and their associated inputs/outputs is reflected in the NESHIE code book. This code book was formulated with the construction of the REDCap database and allows the interpretation of the data. Since REDCap is used, a data dictionary is available for each iteration of the database, as are CDISC ODM files. File naming conventions for clinical data is not used but is applied to molecular data in accordance with external collaborators associated with sample analysis, funder guidelines and other standard practices. Sufficient metadata exists for data interpretation, and CRF version details are captured to the REDCap system for each participant record. FAIR principals are applied as reasonably as possible due to the vulnerability of the participants/study population.

With regard to data sharing and associated standards in the NESHIE study, while these typically do not apply to the clinical data being collected since a single condition is under investigation, such standards may be relevant for other studies and are important for consideration (e.g., SNOMED/CT or ICD-10/11). Additionally, there are currently no formal reporting standards to describe the metadata at a dataset level. In relation to sample data that would be shared as part of the NESHIE study, standard gene identifiers such as HGNC IDs and rsIDs are used for the genetic and transcriptomic data, while gene annotations (e.g., Gene Ontology) are used for proteomic and metabolomic data. All magnetic resonance image files are generated in a DICOM format.

#### 2.3.5 Data storage and database security

Research projects consisting of multi-sectoral collaborators (including the community or institutional ethics committees) may require significant data protection to prevent data access breaches that may violate participants’ rights to privacy (Hardy et al., 2016). Besides noting data security structures in place for a study, the DMP should also explain the storage and protection of the data during the lifecycle of the project (Michener, 2015). Protection of data must include a guide as to how many copies and what format copies of data should take. Copies of data can be in hard- and/or soft-copy format. The storage location of copies should be made known to relevant study representatives; access to data copies should be restricted according to the role and responsibility each research associate holds within the study. A regular backup of the data should also be scheduled.

With regard to the NESHIE study, a three-layered approach to backing up server-hosted data has been taken. First, there is a daily back-up of captured data to a local server. Second, there is a weekly back-up of the entire data ecosystem (which includes uploaded documents), also to a local server. Thirdly, there is a weekly back-up of the entire data ecosystem to a local server that is independent of the server(s) used for points one and two. This backup schedule is consistently tested to ensure the retrieval of the stored data files. Log files are received and viewed by IT specialists to ensure that these systems operate as expected. Such information may be presented in different ways in the DMP but should be appropriate and relevant for each individual study. The flow of storage and sharing of samples and data for analysis purposes in the NESHIE study is detailed in the DMP using a figure, while security considerations regarding the NESHIE electronic database platforms is described in a table format. A template of the key security considerations used for this latter purpose is summarized in Table 6. Internal review of security settings are usually checked and confirmed at the point of user creation and assignment to projects. In REDCap, this is tested by utilizing the ‘view project as’ function, while LogicalDOC facilitates a visualized output of user security settings. In both instances, these functions are reserved only to those individuals within the study assigned with admin rights.

**TABLE 6:** Technical aspects of electronic platforms to report in a study DMP.

	Electronic platform name
Installed version	Version # (Date: XXX)
Updated version	Version # (Date: XXX)
Overview description	E.g., Web-based interface for user/server interaction; Document repository? (‘Yes’, ‘No’, ‘Limited’?); Clinical research platform for data captureetc.
Technical description	E.g., TomCat-based document storage and management; PHP-Apache-MySQL-based clinical research platformetc.
Security	E.g., Secure encrypted website using LetsEncrypt certificates; Dependent on IT infrastructure and environment of host server; Multi-level securityetc.
Servers	Example: Number of servers: web data and database Number of servers: back-up All servers hosted: [insert location name(s) of each server]
Web server requirements	E.g., TomCat 8.5 or higher; PHP 5.3.0 or higher
Database server requirements	E.g., MySQL 5.0+; MariaDB 5.1+
SMTP email server	E.g., Configuration with PHP required on web server
File server	E.g., Files stored on file system of database server; may be separate from database server; files stored behind firewall location for study; WebDAV application available when firewall storage is not used
SSL certificate required	Yes/No
File or Data storage method	Ext4 file system; MySQL back-end with PHP front-end
3rd party server access or use	Not applicable/Applicable [list 3rd party server access/use]
User privileges	Multi-level user privileges at system level (broad or constricted/limited); System administrator assigns initial user privileges at onset
Authentication	Validation of end-users required (State specific authentication methods if applicable)
Auto-logout function	Yes/No
Logging and audit trail	Yes/No
File or Data import function	Yes/No (Describe if ‘Yes’)
File or Data export function	Yes/No (Describe if ‘Yes’)
File or Data interoperability	Import files or folders stored at remote locations using FTP, SFTP, *etc.*,; API via API tokens; data import and export; Dynamic Data Pull (DDP) via web service; data import only

Due to the sensitivity of the data collected for this study, cloud storage of our clinical data is not being used. However, data repositories such as the European Genome Phenome Archive (EGA) are being considered for sample-associated data storage under contract and accessibility by way of a data access committee (DAC). Data transfer agreements would stipulate data transfer conditions, including those pertinent to POPIA regulations.

### 2.4 Sample/data access and sharing

#### 2.4.1 Sample/data sharing intentions and access requirements

Even though good data protection and sharing policies might be in place, this does not mean that data will not be shared (Michener, 2015). Data can be disseminated in a passive or active way. Passive sharing includes posting data on a website or emailing it. Active dissemination, which is preferred, includes submitting the data to an open repository or archive, or publishing the data as articles/Supplementary Material in peer-reviewed research or data journals. Data can be correctly cited by using the guidelines and mechanisms provided by journals and repositories, for example, DOIs. This ensures that researchers are accredited for their data products. Furthermore, data will be more user-friendly and interpretable if it is distributed through standard, non-proprietary approaches. The data should then also include metadata and code which will enable data processing. Licensing and copyright may involve allocating an identifier that is unique to a dataset (Fadlelmola et al., 2021). This will facilitate data discovery and any legal issues when reusing the data.

When writing about the sharing of data in the DMP, it is important to consider data responsibility, accountability and authority (Fadlelmola et al., 2021). These considerations are usually stated in data protection policies such as POPIA or GDPR. Protecting the rights of research participants, especially those who are from vulnerable populations, has become crucial. This has resulted in the adoption of data protection policies globally to provide this framework for both the research participants and data users. This is especially relevant in genomic research as individual genomes are considered personally identifiable information even after participant anonymization (Fadlelmola et al., 2021).

As described by Michener (2015), DMPs should include policy statements regarding the management and sharing of data. These should bear, at the very least, reference to:

• Licensing or sharing arrangements about the use of pre-existing materials;
• Arrangements for retaining, licensing, sharing, and embargoing data, code, and other materials; and
• Legal and ethical restrictions on access and use of sensitive data from research participants.

The level of data access should be determined by a study’s management team with sample/data access and sharing plans approved by an institutional ethics committee/board and/or study-associated data access committee (Fadlelmola et al., 2021). The DMP explains whether access is limited or open, who has access to the study-related data, and whether access to the data has to be approved by a DAC. This applies to the NESHIE study where only individuals associated with the study or duly appointed study-associated representatives have access to the data and samples; Table 7 provides a template of how this can be presented within a DMP. Metadata export for the NESHIE study can currently only be done by a limited number of people when necessary for sample/image analysis.

**TABLE 7:** An example of data and sample access specifications.

Clinical data	
Where is the data stored?	
Who does the data belong to?	
Who/what institution has access to datasets for analysis purposes?	
Who/what institution is permitted to perform the analysis of data?	
What platform(s) will be used to perform the data analysis?	
REC reference institution/approval numbers	

Study-associated samples: Specific (e.g. Blood)	
Where are samples stored:	
*Once collected?*	
*After analysis?*	
Who is permitted to perform the sample nucleic acid isolations?	
Who/what institution is permitted to perform the nucleic sequencing?	
Who/what institution is permitted to perform the analysis of the nucleic acid sequences?	

Importantly, local legislation may require that additional sample and data access-related documents be noted within a DMP. In South Africa, sample analyses not being performed in the country require an accompanying export permit. A material transfer agreement (MTA) between the study’s host institution and the representative organization of another country is required as part of the application for an export permit. Memorandums of understanding are established as supporting documents to MTAs and may describe sample and/or data processing requirements in more detail. This principal similarly applies to the transfer of data and the need for signed data transfer agreements (DTAs). DS-I Africa Law Research Group from the University of KwaZulu-Natal recently presented a DTA template that is an excellent resource for the provision of DTAs in the South African research context (Swales et al., 2023).

### 2.5 Data archiving

#### 2.5.1 Retention period

The archive period of data for research purposes should be mentioned in a DMP. Sample and data retention periods are usually stipulated in the institutional regulations/policies and in funder guidelines. The NESHIE data will be archived for at least 15 years from completion of the study according to UP regulations. When access to data is required for analytical purposes, it should be able to be uncompressed, unencrypted, and decoded from standard character encodings such as 16-bit Unicode Transformation Format or UTF-16 (Michener, 2015).

#### 2.5.2 Repositories

Different digital data repositories are available that provide secure and remote access to their web-based platforms (Antonio et al., 2020). These data repositories store large data sets and are supported by funders and government agencies. This facilitates data sharing across research teams or sharing datasets for a single study where a formal data access application process has been approved. Academic institutions may also provide data repositories that support private and selectively restrictive institutional access to multiple research studies. When selecting a data repository, there are three considerations: physical features (servers and hardware), technical features (software), and administrative features (personnel requirements and support, policies, security and data access). The key function of a data repository is to support secure data management for geographically dispersed research institutions and to provide secure data access, storage, and sharing (Antonio et al., 2020).

If data is archived in an unsecure location for a long-term period, both the researchers and others may not be able to use it as it becomes inaccessible (Michener, 2015). The description of the storage and preservation of data are therefore essential to any good DMP. In other words, three questions have to be considered (Michener, 2015):

1) “How long will the data be accessible?”;
2) “How will data be stored and protected over the duration of the project?”; and
3) “How will data be preserved and made available for future use?”.

Several factors are involved when answering the first question. First, research funders or institutions may have specific requirements. Second, the core value of the data should be considered in relation to the ease with which it can be generated independently of the initial study. To answer question 3, a robust solution may be necessary to access data 20 years after the finalization of a project.

Funders and research institutions may have identified appropriate data repositories for specific research areas. Certain disciplines maintain specific repositories, for example, GenBank is a repository for nucleotide sequence data. Universities may host institutional repositories or general science data repositories, for example, Figshare^13^. Alternatively, there are the Registry of Research Data Repositories^14^ and BioSharing^15^ which are discipline-specific and general repositories via online catalogues. The DMP should note the policies of the selected repository, specifically for data privacy and security (Fadlelmola et al., 2021).

Funders may provide a list of approved repositories for data archiving, but if these repositories do not have the required functionalities or compliance to participant consent conditions, researchers may request the use of other repositories. In terms of repositories for the NESHIE study, several repositories recommended by one of the funding bodies were considered and included: MassIVE, Panorama, Pride (Proteomics Identifications Database) and Metabolomics Workbench. However, the UP REC requires that all NESHIE sample-generated data be archived in a repository that is safeguarded by a DAC. Therefore, with approval from the funding body, it was decided that the EGA will be predominantly used for the purposes of the majority of the NESHIE study ‘omics’ data. NESHIE clinical data is not available for public access.

#### 2.5.3 Future use of data and the data access committee (DAC)

The DMP should provide details regarding how data may be used in the future, for instance, in publications, industry involvement or commercialization. Consideration should also be given to potential funding and/or publication requirements regarding data access. The participant informed consent form should clearly indicate any envisaged future use of data and must serve as the foundation for future access to and use of study-related data.

If applicable, the DMP should provide a description of a study-related DAC. This description explains the role of the DAC and the documents that govern the terms and conditions on which access is granted to the data by the DAC. Access management through a DAC endorses the benefits of data sharing while diminishing the risk of uncontrolled access to study data that is generated from vulnerable/at risk study populations for uses that may fall outside of the purview and restrictions established by the study and its associated consent conditions. Requests are approved or rejected by the DAC rather than having open access without restrictions (Cheah and Piasecki, 2020). In other words, the DAC regulates access to all data generated from a funded research project. The DAC’s purpose includes both the promotion of data sharing and the protection of research participants and their communities, researchers, and research institutions. The establishment of the DAC should adhere to institutional and legal policies together with clear distinctions of responsibility, terms of reference and membership (Cheah and Piasecki, 2020). To accomplish its role, a DAC’s members should represent relevant areas of expertise. Some members may be independent as this will address the issues where conflicts of interest may be involved. The application procedure for data access should be transparent, consistent and simple. The data sharing policies of institutions should provide guidelines for the review process while independent DACs should be guided by pre-agreed terms. Elements of the review should include the applicants, what the objectives of data reuse are, which data are requested, and the potential benefits and risks involved. In order to monitor the flow of data requests and the decisions made thereto, one could either design a custom database or use an equivalent platform in a public domain, such as Resource Entitlement Management System (REMS)^16^.

Sustainability of a fully-functional DAC is a challenge faced in many studies. This should be specifically addressed and may need to be institutional in order to be sustainable. At the very least, it should not be constituted by study members that form part of a mobile community. Nevertheless, a lack of resources/support at an institutional level for the sustainability of a DAC should not prohibit a study from pursuing such endeavors. This does however create additional expectations for the study to appropriately plan and budget for the establishment and maintenance of a DAC. It is a recognized imperfect system but is often the only means through which ‘omics’ data generated on vulnerable populations may be disseminated. It is therefore in the best interest of studies involving such populations to carefully consider the long-term requirements of a DAC during the development phase of the study to ensure that an appropriate strategy may be put in place for its ultimate success. Within the NESHIE study, the onus falls on the study to provide the resources and support for the establishment and maintenance of the DAC.

## 3 Conclusion

A data management plan should provide a user-friendly road map that guides and explains the governance of data throughout the duration of a research project and also after the conclusion thereof. The DMP template presented here, as drafted from the NESHIE study DMP, provides a thorough design or framework that will require approval by reviewers and funders, and that can be applied to a research study or a clinical trial being conducted in vulnerable populations and/or employs muti-omics analysis methods to achieve the study aims. While there should be limited duplication between the protocol and DMP, the DMP does not need to be a self-standing document if the inclusion of protocol information is able to reasonably add value and context to the content of the DMP. The template can nevertheless be adjusted to reference the relevant protocol section(s) where necessary, should a self-standing DMP be required.

The NESHIE DMP remains a dynamic, living document, that will continue to change as legal, ethical, funding, publishing, resource-related and other factors evolve. As an example, the study’s approach to informed consent and the associated documentation and data storage was adjusted with the implementation of POPIA. As a second example, data deposition to a repository was initially not approved as part of the initial ethics approval process. Data deposition to a repository has subsequently been approved, but with important considerations regarding data security given the vulnerability of participants enrolled into this study. As a final example, the NESHIE DMP did not need to make consideration for magnetic resonance image storage at the study’s onset. This changed with the introduction of a portable ultra low-field magnetic resonance which required careful consideration and adjustment to the DMP regarding image acquisition and data transfer. It is therefore unsurprising that this DMP will continue to be adjusted as the project continues to evolve.

Nevertheless, it is clear that DMPs are adaptable in part or in whole and can be applied to successive research studies or clinical trials. An accessible DMP may support researchers and funders in data discovery and future collaborators, provide education on data management, and may monitor compliance with policies and regulations. In considering the nature of DMPs, future work will therefore include describing the lessons learnt throughout the NESHIE study in relation to adjustments to the DMP, particularly regarding the areas prone to change. Future work will also focus on creating a machine-readable DMP version.

## Data Availability

The original contributions presented in the study are included in the article/Supplementary Material, further inquiries can be directed to the corresponding author.

## Footnotes

1 Stanford University. (2023). Data management plans. https://doresearch.stanford.edu/topics/manage-research-data [Accessed 28 August 2023]

2 DMPonline. (2023). Plan to make data work for you. https://dmponline.dcc.ac.uk/[Accessed 5 September 2022]

3 DMPTool. (2023). Create Data Management Plans that meet requirements and promote your research. https://dmptool.org/[Accessed 5 September 2022]

4 GO FAIR. (2023). FAIR Principles. https://www.go-fair.org/fair-principles/[Accessed 6 September 2022]

5 News24. (2023). State hospitals pay huge legal claims for cerebral palsy, but new study makes surprising claims. https://www.news24.com/fin24/companies/state-hospitals-pay-huge-legal-claims-for-cerebral-palsy-but-new-study-makes-surprising-claims-20210622 [Accessed 5 September 2023]

6 Daily Maverick. (2023). Cerebral palsy in South Africa—medical negligence is just one of many causes. https://www.dailymaverick.co.za/article/2022-07-04-cerebral-palsy-in-south-africa- medical-negligence-is-just-one-of-many-causes/[Accessed 15 March 2023]

7 News24. (2023). Landmark medical negligence ruling orders SA hospitals to treat—not pay—victim. https://www.news24.com/fin24/companies/landmark-medical-negligence-ruling-orders-sa-hospitals-to-treat- not-pay-victim-20230213 [Accessed 5 September 2023]

8 Society for Clinical Data Management. (2023). Data Management Plan.https://scdm.org/wp-content/uploads/2019/12/GCDMP- Data-Management-Plan-2019-Edition.pdf [Accessed 15 March 2023]

9 University of Toronto. (2023). Data Management Plans.https://onesearch.library.utoronto.ca/researchdata/data-management-plans [Accessed 3 August 2023]

10 DCC. (2023). Data Management Plans. https://www.dcc.ac.uk/resources/data-management-plans [Accessed 3 August 2023]

11 SAHPRA. (2023). South African Good Clinical Practice: Clinical Trial Guidelines. https://www.sahpra.org.za/wp-content/uploads/2021/06/SA-GCP-2020_Final.pdf [Accessed 24 July 2023]

12 NCBI. (2023). Guideline for Human Gene Nomenclature. https:// www.ncbi.nlm.nih.gov/pmc/articles/PMC7494048/#:∼:text=Each%20gene%20is%20assigned%20a,or%20%E2%80%9CG%E2%80%9D%20for%20gene [Accessed 24 July 2023]

13 Figshare. (2023). Figshare as a data repository. https://figshare.com/[Accessed 9 November 2022]

14 Re3data. (2023). Registry of Research Data Repositories. https://www.re3data.org/ [Accessed 9 November 2022]

15 BioSharing Network. (2023). Blood donor repository. https://bio-sharing.org/ [Accessed 9 November 2022]

16 GitHub. (2023). REMS. https://github.com/CSCfi/rems [Accessed 5 September 2023]
