# Supplementary material for: A data management plan for the NESHIE observational study
Source: Front Genet. 2023 Dec 6;14:1273975. doi: 10.3389/fgene.2023.1273975 (PMC10734687; doi:10.3389/fgene.2023.1273975)
Supplement: Supplementary file 1 [file Table1.docx]

Supplementary Material

Template

Title of research project

**Document name (E.g. Data Management Plan)**

Document Version number (E.g. Version 1.0)

| Name of organization where research is being done (list if multiple)  Date current DMP version came into effect  Other administrative details (E.g. IRB/REC approval numbers) | E.g. University of Pretoria  E.g. 7 April 2023  E.g. 123/2023 |
| --- | --- |

| DMP Prepared by* | *Name & Surname* |
| --- | --- |
| DMP Reviewed by* | *Name & Surname* |
| DMP Approved by* | *Name & Surname* |

*List all individuals if more than one person is involved in any part of this

**Revision History:**

| **Version Number** | **Date** | **Author(s)** | **Authorized by** | **Description of amendments made** |
| --- | --- | --- | --- | --- |
| E.g. 1 | E.g. 7 March 2022 | Full Name | Full Name | E.g. Section # - IP agreements (updated)  Annexure: Agreements (updated) |

**Table of Contents**

[1 List of abbreviations 3](#_Toc141278856)

[2 Definitions 3](#_Toc141278857)

[3 Scope 3](#_Toc141278858)

[4 Protocol Summary or Introduction 3](#_Toc141278859)

[5 Core elements of the DMP 3](#_Toc141278860)

[5.1 Project and Sample/Data collection 3](#_Toc141278861)

[5.2 Data Characteristics 10](#_Toc141278862)

[5.3 Samples/Data Storage and Security 15](#_Toc141278863)

[5.4 Sample/Data Access and Sharing 18](#_Toc141278864)

[5.5 Data Archiving 19](#_Toc141278865)

[6 Annexures 20](#_Toc141278866)

# List of abbreviations

This section should include a list of abbreviations, specific to the DMP. For example:

**CRF**: Case Report Form

**DMP**: Data Management Plan

**DAC**: Data Access Committee

Definitions

This section should include standard definitions of the terms used in the DMP that may be misinterpreted or misunderstood. For example:

- **Cord Blood:** Blood from the umbilical cord.
- **REDCap:** Server-based Research Electronic Data Capture platform used for the collection and management of patient data.

# Scope

Briefly describe the application of the DMP in the research project, as well as policies and regulations to which the project complies.

# Protocol Summary or Introduction

Provide a short summary or broad overview of the study protocol and variables for critical data analysis.

# Core elements of the DMP

## Project and Sample/Data collection

The following sections describe the management of the research project and the samples/data collection process.

### Research team and training

Specify the research team responsible for the data management activities in the project. Also describe and list the study-specific training for the research team. The following may be tabulated in the DMP as indicated below:

- Who is collecting the samples/data? E.g. scientists, research assistants, etc.
- What are their responsibilities? E.g. collection and entry of data, quality control, etc.
- What are the training requirements for the research team? E.g. study protocol, sample collection, etc.
- When is the training provided? E.g. prior to study onset, etc.
- Is the training recorded? E.g. study-associated logbook or file, etc.

**Research team specifications**

|  | **Full name(s) & initials of responsible party/parties** | **Study associated sample/data responsibility** | | | | | | | | | | **Date responsibility assigned** | **Date responsibility ended** | **Signature (optional; study** |
| --- | --- | --- | --- | --- | --- | --- | --- | --- | --- | --- | --- | --- | --- | --- |
| **Project Leads** | **Researcher One (R.O.)** |  | **1**  **2**  **3** |  | **4**  **5**  **6** |  | **7**  **8**  **9** |  | **10**  **11**  **12** |  | **13**  **14**  **15** |  |  |  |
|  | **Clinician One (C.T.)** |  | **1**  **2**  **3** |  | **4**  **5**  **6** |  | **7**  **8**  **9** |  | **10**  **11**  **12** |  | **13**  **14**  **15** |  |  |  |
| **Project Site Support** | **Site Obstetrician One (S.O.O.)** |  | **1**  **2**  **3** |  | **4**  **5**  **6** |  | **7**  **8**  **9** |  | **10**  **11**  **12** |  | **13**  **14**  **15** |  |  |  |
| **Biomarker & Clinical Trial Component** | **Clinical Trial Officer One (C.T.O.O.)** |  | **1**  **2**  **3** |  | **4**  **5**  **6** |  | **7**  **8**  **9** |  | **10**  **11**  **12** |  | **13**  **14**  **15** |  |  |  |
| **Electronic Data Capture Platform** | **IT Support One (I.T.S.O)** |  | **1**  **2**  **3** |  | **4**  **5**  **6** |  | **7**  **8**  **9** |  | **10**  **11**  **12** |  | **13**  **14**  **15** |  |  |  |
| **Study Appointees** | **Study Appointee One (S.A.O.)** |  | **1**  **2**  **3** |  | **4**  **5**  **6** |  | **7**  **8**  **9** |  | **10**  **11**  **12** |  | **13**  **14**  **15** |  |  |  |
| **[Specify study role]** | **[Specify full name]** |  | **1**  **2**  **3** |  | **4**  **5**  **6** |  | **7**  **8**  **9** |  | **10**  **11**  **12** |  | **13**  **14**  **15** |  |  |  |

**Study associated responsibility codes:**

| **01** | Sample collection | **06** | Data collection to CRF | **11** | [Specify study specific responsibility] |
| --- | --- | --- | --- | --- | --- |
| **02** | Sample shipment: Storage | **07** | Data capture to electronic database | **12** | [Specify study specific responsibility] |
| **03** | Sample shipment: Analysis | **08** | Data validation | **13** | [Specify study specific responsibility] |
| **04** | Sample storage: On-site | **09** | Record locking | **14** | [Specify study specific responsibility] |
| **05** | Sample storage: Off-site | **10** | Database locking | **15** | [Specify study specific responsibility] |

**Training requirements:**

|  | **Protocol** | **SOP: 1** | **SOP: 2** | **ICF** | **Comm. Engage** | **CRF** | **Data capture** | **Sample collection** | **Sample shipment** | **[Specify study doc.]** | **[specify study doc.]** | **[specify study doc.]** | **[specify study doc.]** | **[specify study doc.]** | **[specify study doc.]** | **[specify study doc.]** | **[specify study doc.]** | **[specify study doc.]** |
| --- | --- | --- | --- | --- | --- | --- | --- | --- | --- | --- | --- | --- | --- | --- | --- | --- | --- | --- |
| **Research Scientists** |  |  |  |  |  |  |  |  |  |  |  |  |  |  |  |  |  |  |
| **Research Assistants** |  |  |  |  |  |  |  |  |  |  |  |  |  |  |  |  |  |  |
| **Clinicians** |  |  |  |  |  |  |  |  |  |  |  |  |  |  |  |  |  |  |
| **Allied health workers (e.g. nurses)** |  |  |  |  |  |  |  |  |  |  |  |  |  |  |  |  |  |  |
| **QC officers: Data** |  |  |  |  |  |  |  |  |  |  |  |  |  |  |  |  |  |  |
| **[Specify study role]** |  |  |  |  |  |  |  |  |  |  |  |  |  |  |  |  |  |  |

|  | **Protocol** | **SOP: 1** | **SOP: 2** | **ICF** | **Comm. Engage** | **CRF** | **Data capture** | **Sample collection** | **Sample shipment** | **[specify study doc.]** | **[specify study doc.]** | **[specify study doc.]** | **[specify study doc.]** | **[specify study doc.]** | **[Specify study doc.]** | **[Specify study doc.]** | **[Specify study doc.]** | **[Specify study doc.]** |
| --- | --- | --- | --- | --- | --- | --- | --- | --- | --- | --- | --- | --- | --- | --- | --- | --- | --- | --- |
| **Training: Prior to study onset** |  |  |  |  |  |  |  |  |  |  |  |  |  |  |  |  |  |  |
| **Training: After study onset** |  |  |  |  |  |  |  |  |  |  |  |  |  |  |  |  |  |  |
| **Continuous training** |  |  |  |  |  |  |  |  |  |  |  |  |  |  |  |  |  |  |
| **Once-off training** |  |  |  |  |  |  |  |  |  |  |  |  |  |  |  |  |  |  |
| **In-person training** |  |  |  |  |  |  |  |  |  |  |  |  |  |  |  |  |  |  |
| **Online training** |  |  |  |  |  |  |  |  |  |  |  |  |  |  |  |  |  |  |

**Training Records: These documents/recordings (if applicable) will be stored [Specify storage location]**

### Purpose and strategy of sample/data collection

Describe what samples and data are to be collected/generated, the source of the samples and data, as well as the future analysis strategy of the data. An overview of the sample and data collection strategy can be tabulated (with tick boxes), for example:

- What is the DMP focus area for samples and data? E.g. sample and data characteristics, data confidentiality and anonymization, data application, etc.
- What samples are collected? E.g. blood, tissue, etc.
- What is the source of the samples? E.g. whole blood, fresh/frozen, etc.
- What is the volume/weight for each sample to be collected? E.g. µL or mL, etc.
- How many samples will be collected? E.g. single or multiple samples, etc.
- When will samples be collected? E.g. prior or during treatment, etc.
- What data are collected? E.g. treatments, imaging, etc.

It is also important to list the aims and objectives (if applicable) of the sample and data collection process in this section of the DMP.

**Overview: Sample and Data Collection Strategy**

**Clinical Data**

**Biological Samples / Data**

CRF  Repository data

Blood  Saliva  Urine

Tissue  DBS  Stool

*Neonatal data*

Treatment data

Follow-up data

*Maternal and/or Paternal data*

Medical history

Antenatal care data

Delivery data

Post-partum data

Placental histology data

*Imaging data*

Ultrasound

MRI scans

CAT scans

**DMP focus area**

**Samples & Clinical Data To Be Used**

Source of [Blood Sample]:  Serum  Plasma  Whole blood

Source of [Tissue Sample]:  Fresh  Frozen  Fixed

Source of [DBS Sample]:  Heel prick  ABG  Peripheral blood

Volume or weight of [Each Sample] to be collected?

- - E.g. 75µL DBS sample, or 3mL blood from consenting adult, etc.

How many samples/sections of each sample type will be collected?

Single sample: [Specify]

Multiple samples: [Specify sample type & quantity]

When will the samples be taken?

Prior to treatment: [Specify sample(s)]

During treatment: [Specify sample(s)]

After treatment: [Specify sample(s)]

**Data confidentiality:**

All data are sensitive/confidential

Mixed degree of data sensitivity

Limited data sensitivity

No data sensitivity / N/A

All data are sensitive/confidential

Mixed degree of data sensitivity

Limited data sensitivity

No data sensitivity / N/A

**Data identifiability:**

Data anonymization

Data pseudonymization

No anonymization needed

N/A (repository data)

Data anonymization

Data pseudonymization

No anonymization needed

N/A (repository data)

**Data application:**

Epidemiological investigation

PheWAS

Specific investigation (state)

GWAS (Specify sample/s)  Transcriptomics (Specify sample/s)

Metabolomics (Specify sample/s)  Proteomics (Specify sample/s)

Microbiomics (Specify sample/s)  Other investigation (State)

**Sample & Data Characteristics**

### Regulatory requirements and informed consent

This section describes the ethics approval obtained for research purposes and may be provided as bullet points or in table format. The following must be recorded for all participating institutions and sites:

- What is the regulatory body’s name? E.g. institutional REC/IRB or site, etc.
- What is the study approval number? E.g. ethics reference number, etc.
- What is the ethics approval date? E.g. date when REC/IRB approved study, etc.

| **Name of regulatory body** | **Study approval number** | **Date application: Submitted** | **Date application: Approved** |
| --- | --- | --- | --- |
|  |  | dd/mmm/yyyy | dd/mmm/yyyy |
|  |  | dd/mmm/yyyy | dd/mmm/yyyy |

Additionally, as study documents may require amendments during the project, a record of changes may be kept, for example, a document tracking log. Although the tracking log does not form part of the DMP, reference to its storage location should be made. The document tracking log may be tabulated as follows:

- Document amendment no., e.g. Amendment 1, etc.
- Document name, e.g. Study protocol, etc.
- Document version in use under current amendment, e.g. Version 1, etc.
- Date of REC/IRB amendment submission
- Date of REC/IRB amendment approval

| **Amendment application number** | **Document type**  **[Specify as relevant for your study]** | **Version in use under this Amendment** | **Date submitted: REC** | **Date approved:**  **REC** |
| --- | --- | --- | --- | --- |
| Amendment # | Protocol | Version # | dd/mmm/yyyy | dd/mmm/yyyy |
| Amendment # | Informed consent form (ICF) | Version # | dd/mmm/yyyy | dd/mmm/yyyy |
| Amendment # | SOP 1 | Version # | dd/mmm/yyyy | dd/mmm/yyyy |
| Amendment # | SOP 2 | Version # | dd/mmm/yyyy | dd/mmm/yyyy |
| Amendment # | Case Report Form (CRF) | Version # | dd/mmm/yyyy | dd/mmm/yyyy |

Other information that should be described in this section:

- Inclusion and exclusion criteria for participation in the study. Some funders or other regulatory bodies may require that these criteria should be described in the DMP. However, a reference to the relevant study document in the DMP may simplify this issue. The details may be provided in text or table format. Usually, this information contextualize the informed consent process in the study.
- A reference to the consent documents and where they may be found.
- Description of the consent process and associated vetting thereof. This may include the quality control process to ensure the validity of each signed consent form from participants.

### Quality Assurance (QA) and Quality Control (QC) processes

This section in the DMP should describe the following:

- Is there a QA/QC plan?

If so:

- How will data be checked to ensure high quality?
- Who will be responsible for performing the various QA/QC tasks?
- When/How frequently will these tasks be performed?
- What are the potential problem areas?
- Are there contingency plans in place?

Templates for the purpose of QA/QC should be reflected in the DMP. An overview of the QA/QC process may be provided as a figure in the DMP.

| How will data be checked? | Manually | Semi-automated | | | Fully automated | |
| --- | --- | --- | --- | --- | --- | --- |
| Who will be responsible for:  QC of Clinical Data | [Specify full name of responsible individual(s)] | | | | | |
| Who will be responsible for:  QC of [Specify specific QC step] | [Specify full name of responsible individual(s)] | | | | | |
| When/How frequently will QC be performed: Clinical data | Once-off | | | Continually | | |
|  | Daily  Weekly  Monthly | | Quarterly  Bi-annually  Annually | | | Mid-study  End of study |
| When/How frequently will QC be performed: [Specify specific QC step] | Once-off | | | Continually | | |
|  | Daily  Weekly  Monthly | | Quarterly  Bi-annually  Annually | | | Mid-study  End of study |
| What are the potential problem areas? |  | | | | | |
| Are there contingency plans in place? |  | | | | | |

### Ownership

In this section of the DMP the following is described:

- Ownership of the research data;
- Ownership of the data collected at the research sites;
- The DMP refers only to a working group document that includes a list of people involved in the research project. This name list is confidential and should not be included in the text of the DMP;
- A list of intellectual property agreements in place, e.g. MTAs, MOUs, etc. These documents may also be listed in the Annexure but kept separately from the DMP owing to confidentiality.
- Whether any commercialization or industry involvement has been considered;
- The funders of the research project and their requirements for data management;
- Publication expectations and acknowledgements; and
- If the research study is a collaborative project, all collaborators and their roles in the study should be provided in the DMP.

### Auditing

The DMP should either include the details of an audit plan for a clinical trial or refer to the relevant SOP for a research project. When a research project does not have a formal audit plan, the manner of data validation should be explained.

### Website

If a website has been created for a research project or clinical trial, the details should be provided in the DMP, for example:

- The status of the website, e.g. under development or functional, etc.;
- Provide a website address if functional;
- Whether the website is private or open to the public;
- The regulation of the website; and
- The aim of the website, e.g. current research and publications, etc.

## Data Characteristics

The following sections describe the data collected or generated during the research.

### Data collection sheets

This section in the DMP should describe the following:

- Development of the data/sample collection sheets (case report forms or CRFs);
- General guidelines for the completion of these collection sheets;
- Amendments made to the collection sheets; and
- Recordkeeping practices.

### Data submission

This section describes how the data is submitted, for example:

- Whether it is submitted manually or electronically, or even both;
- The tools used to generate the data (e.g. standardized CRFs and electronic data capture platforms) and submit the data (e.g. electronic or hard copy). This can be provided in table format;
- Whether the submission mode is compliant with the guidelines and policies applicable for the research being conducted (e.g. enforcing Good Clinical Practice guidelines in clinical trials);
- The submission of anonymized and identifying data; and
- Data verification.

| **Generated Document/Data** | **Submission Type** | **Anonymization status** | **[Specify standard] Compliant?** | **[Specify standard] Compliant?** | **Data to be verified?** |
| --- | --- | --- | --- | --- | --- |
| Informed Consent Forms (ICFs) | Electronic: Site & Study records | Yes; fully anonymized  Yes; pseudonymized  No; fully identifiable | Yes; fully  Yes; partially  No; not compliant | Yes; fully  Yes; partially  No; not compliant | Yes; fully  Yes; partially  No; not applicable |
|  | Hard-copy: Site records | Yes; fully anonymized  Yes; pseudonymized  No; fully identifiable | Yes; fully  Yes; partially  No; not compliant | Yes; fully  Yes; partially  No; not compliant | Yes; fully  Yes; partially  No; not applicable |
| Community Engagement Form(s) | Electronic: Site & Study records | Yes; fully anonymized  Yes; pseudonymized  No; fully identifiable | Yes; fully  Yes; partially  No; not compliant | Yes; fully  Yes; partially  No; not compliant | Yes; fully  Yes; partially  No; not applicable |
|  | Hard-copy: Site records | Yes; fully anonymized  Yes; pseudonymized  No; fully identifiable | Yes; fully  Yes; partially  No; not compliant | Yes; fully  Yes; partially  No; not compliant | Yes; fully  Yes; partially  No; not applicable |
|  | Hard-copy: Study Master File | Yes; fully anonymized  Yes; pseudonymized  No; fully identifiable | Yes; fully  Yes; partially  No; not compliant | Yes; fully  Yes; partially  No; not compliant | Yes; fully  Yes; partially  No; not applicable |
| Clinical data storage | Electronic: Site & Study records | Yes; fully anonymized  Yes; pseudonymized  No; fully identifiable | Yes; fully  Yes; partially  No; not compliant | Yes; fully  Yes; partially  No; not compliant | Yes; fully  Yes; partially  No; not applicable |
|  | Hard-copy: Site records | Yes; fully anonymized  Yes; pseudonymized  No; fully identifiable | Yes; fully  Yes; partially  No; not compliant | Yes; fully  Yes; partially  No; not compliant | Yes; fully  Yes; partially  No; not applicable |
|  | Hard-copy: Study Master File | Yes; fully anonymized  Yes; pseudonymized  No; fully identifiable | Yes; fully  Yes; partially  No; not compliant | Yes; fully  Yes; partially  No; not compliant | Yes; fully  Yes; partially  No; not applicable |

| **Generated Document/Data** | **Submission Type** | **Anonymization status** | **[Specify standard] Compliant?** | **[Specify standard] Compliant?** | **Data to be verified?** |
| --- | --- | --- | --- | --- | --- |
| Basic data for sample storage | Electronic: Sample storage facility | Yes; fully anonymized  Yes; pseudonymized  No; fully identifiable | Yes; fully  Yes; partially  No; not compliant | Yes; fully  Yes; partially  No; not compliant | Yes; fully  Yes; partially  No; not applicable |
|  | Electronic: Site & Study records | Yes; fully anonymized  Yes; pseudonymized  No; fully identifiable | Yes; fully  Yes; partially  No; not compliant | Yes; fully  Yes; partially  No; not compliant | Yes; fully  Yes; partially  No; not applicable |
|  | Hard-copy: Site records | Yes; fully anonymized  Yes; pseudonymized  No; fully identifiable | Yes; fully  Yes; partially  No; not compliant | Yes; fully  Yes; partially  No; not compliant | Yes; fully  Yes; partially  No; not applicable |
|  | Hard-copy: Study Master File | Yes; fully anonymized  Yes; pseudonymized  No; fully identifiable | Yes; fully  Yes; partially  No; not compliant | Yes; fully  Yes; partially  No; not compliant | Yes; fully  Yes; partially  No; not applicable |
| Molecular data storage | Electronic: Sample analysis facility | Yes; fully anonymized  Yes; pseudonymized  No; fully identifiable | Yes; fully  Yes; partially  No; not compliant | Yes; fully  Yes; partially  No; not compliant | Yes; fully  Yes; partially  No; not applicable |
|  | Electronic: Site & Study records | Yes; fully anonymized  Yes; pseudonymized  No; fully identifiable | Yes; fully  Yes; partially  No; not compliant | Yes; fully  Yes; partially  No; not compliant | Yes; fully  Yes; partially  No; not applicable |
|  | Hard-copy: Site records | Yes; fully anonymized  Yes; pseudonymized  No; fully identifiable | Yes; fully  Yes; partially  No; not compliant | Yes; fully  Yes; partially  No; not compliant | Yes; fully  Yes; partially  No; not applicable |
|  | Hard-copy: Study Master File | Yes; fully anonymized  Yes; pseudonymized  No; fully identifiable | Yes; fully  Yes; partially  No; not compliant | Yes; fully  Yes; partially  No; not compliant | Yes; fully  Yes; partially  No; not applicable |

### Sensitive and confidential data

The DMP should describe how sensitive or confidential information will be collected, protected and used.

- Are the research participants from a vulnerable population/community?
- Who has access to this information for QA/QC purposes?
- How, when and how frequently will this information be accessed?

| Are the research participants from a vulnerable population/community? | | | Yes | No |
| --- | --- | --- | --- | --- |
| If ‘Yes’, specify population(s)/community type(s) (E.g. neonates) | | |  | |
|  | | | | |
| Who has access to sensitive data for QA/QC purposes? | | | | |
| Full name: |  | | | |
| QA/QC data |  | | | |
| Data access frequency | Daily | Weekly | Monthly | Mid-study |
|  | Quarterly | Bi-annually | Annually | End of study |
|  | Other: Describe |  | |  |
| Full name: |  | | | |
| QA/QC data |  | | | |
| Data access frequency | Daily | Weekly | Monthly | Mid-study |
|  | Quarterly | Bi-annually | Annually | End of study |
|  | Other: Describe |  | |  |

- How are research findings reported to prevent the identification of individuals?

### Data identifiability

In research studies or clinical trials with human participants, personal information of participants are anonymized to protect their privacy. Therefore, the DMP must explain the coding system used to ensure participant anonymity, for example:

- What coding system is used in the research project? E.g. random alphanumeric code, etc.
- Are the same identifiers used throughout the duration of the research study and applied equally to all data collected? If not, describe the differentiation of these identifiers.
- Are samples anonymized additionally when deposited in a repository, e.g. a biobank?
- Are site-specific master lists maintained to link participant information to the study identifiers?

### Data updates

This section explains how data will be updated or become redundant when revisions are made and subsequent CRFs are produced, for example:

- How often is the DMP revised and updated?
- If data becomes redundant, is information retained from any system when updates are required for recordkeeping and backup purposes?
- Are these data updates reflected in the amendments submitted to an REC/IRB?
- Is their recordkeeping for all changes made to the study documents?
- Are data dictionaries (or equivalent documentation) kept for all changes made on the different eDCP versions?

### Data reporting

The DMP should describe the requirements and frequency of data reporting and this should be reflected in the study-associated timeline, for example:

- When do the data reporting requirements start in the research study?
- When is data reported during the QC process?
- What data is reported internally to the PI and how often?
- How often is data obtained from sample analyses reported?

## Samples/Data Storage and Security

The following sections describe the processes implemented to store the sample/data in a safe and secure manner.

### Database design, creation and maintenance

The DMP should describe the process involved in establishing an electronic database platform which may be summarized in a figure, for example:

| **Phase I** | 1. **Design CRF** | - Design by experts and approved by IRB/REC - Design according to local standard practices and global best practices - Design to include data subsections - Amendments made as necessary |
| --- | --- | --- |
|  | 🡻 | |
|  | 1. **Design eDB on REDCap** | - Establish project settings - Create data collection instruments in line with subsections on CRF (establish branching logic and physical display of variables/data) - Define database events - Assign instruments to events - Enable optional modules and customizations - Establish user rights and permissions |
|  | 🡻 | |
| **Phase II** | 1. **Test eDB** | - Identify errors between CRF and variable being captured on REDCap - Identify branching logic errors - Identify errors when accessing data - Identify errors relating to data permissions or data access groups - Identify other errors that may occur |
|  | 🡻 | |
|  | 1. **Adjust eDB** | - Correct identified errors - Second round of database testing by all parties - Ensure that corrections have not altered functionality of database |
|  | 🡻 | |
|  | 1. **Launch eDB** | - Database moved from developmental mode to production mode - All mock data are removed - Participants’ data are captured - All data are subjected to quality control |
| 🡻 | | |
| **Phase III** | 1. **Maintain eDB** | - Production-mode errors in database are corrected as identified - Flow of data capture is improved based on user feedback - Existing data are retained for recordkeeping purposes |

### Data input and processing

The DMP should explain the guidelines for data entry and processing, for example:

- How will data be processed, e.g. paper-based or electronic, or both?
- How is the CRFs completed?
- How is data entered on the eDCPs?
- Is a single or double data entry method used?
- How is data anomalies/discrepancies managed?
- Are there self-evident corrections to the data and who can make these corrections?
- How is data reconciliation (if applicable) managed?
- What are the requirements for database lock?

### Data format and transformation

The DMP should disclose the study-related data formats. This may easily be explained by using a table with, for example, the following headings:

| **DATA** | **SOFTWARE/SYSTEM USED** | **FILE FORMATS** |
| --- | --- | --- |
| E.g. Genomic | Illumina short read sequencing | FASTQ |

The DMP should also describe the following:

- file format transformations of data
- data export options which includes internal data review and external data review/sharing

### Data standard and metadata

This section in the DMP describes the data standard and the metadata of the research study, for example:

- How will the data be formatted and standardized for present and future use?
- Is there a data dictionary?
- Are file naming conventions applied?
- Is existing metadata sufficient for data interpretation?
- How will different versions be tracked?
- Are FAIR principles applied to the research data?

### Data storage and database security

This section should include details of data storage (e.g. location, user access, etc.) and database security (e.g. security of the equipment, servers and electronic records):

- What are the requirements for data storage (e.g. format and number of copies, etc.)?
- What are the details of database location, user access and platform in operation?
- Who has access to the data copies?
- What are the restrictions for access?
- What is in place to mitigate any data loss?

The flow of storage and sharing of samples and data for analysis purposes may be presented as a figure in the DMP.

- What are the security considerations for electronic database platforms? (E.g. technical aspects of electronic database platforms may be provided in a table format.)

|  | **Electronic platform name:** |
| --- | --- |
| Installed version | Version # (Date: XXX) |
| Updated version | Version # (Date: XXX) |
| Overview description | E.g. Web-based interface for user/server interaction; Document repository? (‘Yes’, ‘No’, ‘Limited’?); Clinical research platform for data capture, etc. |
| Technical description | E.g. TomCat-based document storage and management; PHP-Apache-MySQL-based clinical research platform; etc. |
| Security | E.g. Secure encrypted website using LetsEncrypt certificates; Dependent on IT infrastructure & environment of host server; Multi-level security, etc. |
| Servers | Example:  Number of servers: web data & database  Number of servers: back-up  All servers hosted: [insert location name(s) of each server] |
| Web server requirements | E.g. TomCat 8.5 or higher; PHP 5.3.0 or higher |
| Database server requirements | E.g. MySQL 5.0+; MariaDB 5.1+ |
| SMTP email server | E.g. Configuration with PHP required on web server |
| File server | E.g. Files stored on file system of database server; may be separate from database server; files stored behind firewall location for study; WebDAV application available when firewall storage is not used |
| SSL certificate required | Yes / No |
| File or Data storage method | Ext4 file system; MySQL back-end with PHP front-end |
| 3^rd^ party server access or use | Not applicable / Applicable [list 3^rd^ party server access/use] |
| User privileges | Multi-level user privileges at system level (broad or constricted/limited); System administrator assigns initial user privileges at onset |
| Authentication | Validation of end-users required (State specific authentication methods if applicable) |
| Auto-logout function | Yes / No |
| Logging & audit trail | Yes / No |
| File or Data import function | Yes / No (Describe if ‘Yes’) |
| File or Data export function | Yes / No (Describe if ‘Yes’) |
| File or Data interoperability | Import files or folders stored at remote locations using FTP, SFTP, etc.; API via API tokens; data import & export; Dynamic Data Pull (DDP) via web service; data import only |

## Sample/Data Access and Sharing

The following section describes access control and sharing of the sample/data for future use or reuse.

### Sample/data sharing intentions and access requirements

The DMP should explain the following:

- Who has access to the study-related data?
- Is access limited or open?
- Does data access require approval by a data access committee or not?
- Are additional sample and data access-related documents noted within the DMP? E.g. material/data transfer agreements or memorandums of understanding.

Data and sample access specifications may be tabulated in the DMP.

| **Clinical Data** | |
| --- | --- |
| Where is the data stored? |  |
| Who does the data belong to? |  |
| Who/what institution has access to datasets for analysis purposes? |  |
| Who/what institution is permitted to perform the analysis of data |  |
| What platform(s) will be used to perform the data analysis? |  |
| REC reference institution/approval numbers |  |
| **Study-associated samples: Specific (e.g. Blood)** | |
| Where are samples stored:  *Once collected?*  *After analysis?* |  |
| Who is permitted to perform the sample nucleic acid isolations? |  |
| Who/what institution is permitted to perform the nucleic sequencing? |  |
| Who/what institution is permitted to perform the analysis of the nucleic acid sequences? |  |

## Data Archiving

The following sections describe the long-term storage requirements of the data after the completion of the project to ensure all stakeholder compliance.

### Retention period

Institutional regulations/policies and funder guidelines usually stipulate the retention period for samples and data. Therefore, the DMP should mention the archive period of data for research purposes.

### Repositories

The following should be described in the DMP:

- Has a data repository been selected?
- Does the REC/IRB require that the data repository be safeguarded by a data access committee?
- Does the repository meet the requirements of funders and relevant research institution?
- What are the policies of the selected repository? E.g. for data privacy and security, etc.

### Future use of data and the Data Access Committee (DAC)

The DMP should explain the future use of data with regard to publications, industry involvement or commercialization. The funder guidelines regarding data access (e.g. open access policy) and publication requirements should also be mentioned in the DMP. It is important that the informed consent study documents must indicated whether the research data will be used or accessed in the future after the completion of the research project.

If a data access committee is required for data access, the establishment of this committee must be described in the DMP. This usually refers to the documents governing the terms and conditions on which data access is granted and the role of the committee. These documents should be listed in the Annexures.

# Annexures

The DMP usually refers to documents that are not included in the text, for example, study documents, agreements, policies, guidelines, regulations, etc. Some of these documents are confidential/sensitive and are only available on request. The list of such documents may be tabulated with websites/links that indicate the storage/access location and/or contact person (where applicable). Amendments made to documents that are submitted to an institutional ethics committee/board may be added to the Annexures as a separate document.
